# NSCLC肺部小病灶非均整模式立体定向放疗的应用

**DOI:** 10.3779/j.issn.1009-3419.2015.05.08

**Published:** 2015-05-20

**Authors:** 建昊 耿, 安辉 石, 荣 余, 昊 吴, 广迎 朱

**Affiliations:** 100142 北京，北京大学临床肿瘤学院，北京大学肿瘤医院暨北京市肿瘤防治研究所放射治疗科，恶性肿瘤发病机制及转化研究教育部重点实验室 Department of Radiation Oncology, Peking University Cancer Hospital & Institute, Beijing 100142, China

**Keywords:** 肺肿瘤, 立体定向放射治疗, 非均整模式, Lung neoplasms, Stereotactic body radiation therapy, Flattening filter free

## Abstract

**背景与目的:**

随着放疗技术的快速发展，立体定向放射治疗（stereotactic body radiation therapy, SBRT）已得到广泛应用，且在早期非小细胞肺癌（non-small cell lung cancer, NSCLC）治疗中取得与手术相当的疗效。非均整模式移除了加速器的射野均整器，其剂量率远高于常规均整模式，缩短治疗时间，但目前临床应用较少。本研究旨在探讨非均整模式SBRT治疗的安全性和有效性。

**方法:**

选取2011年12月-2013年12月期间的T1-2N0M0原发NSCLC，术后肺内孤立复发转移，以及Ⅳ期肺内寡转移灶者共31例，予以内在大体肿瘤靶区60 Gy/8 f或48 Gy/4 f的非均整模式SBRT治疗。

**结果:**

非均整模式比常规均整模式明显缩短了治疗时间，靶区剂量相当，且未增加正常组织受量。患者中位随访时间为19.4个月。1年的局部控制、区域控制、远处转移控制、无疾病进展和总生存率分别为96.8%、96.8%、83.9%、77.4%和96.8%。最常见的副反应为放射性肺炎（1级29%，2级3.2%）和胸痛（1级12.9%，2级6.5%），发生率较低。

**结论:**

相比既往常规均整模式，使用非均整模式大分割放射治疗技术治疗肺部小肿瘤是安全、有效的，但长期效果仍需进一步随访、研究。

## Abstract (en)

**Background and objective:**

With the rapid development of technology, stereotactic radiotherapy has been widely used. In a cohort of medically operable non-small cell lung cancer patients receiving stereotactic body radiation therapy (SBRT) survival rates "potentially equivalent to those of surgery" have been reported. Removing the field flattening filter, Clinac is capable of delivering dose rates much higher than conventional linac as well as reducing the treatment time. The goals of this work were to report safety and efficacy of SBRT treatment using a flattening filter-free model for non-small cell lung cancer (NSCLC) with small lesions.

**Methods:**

From December 2011 to December 2013, 31 NSCLC patients who were T1-2N0M0, solitary pulmonary recurrence after surgery, and stage Ⅳ with oligo metastasis were enrolled, receiving SBRT treatment (60 Gy/8 f or 48 Gy/4 f) applying a flattening filter-free model.

**Results:**

Compared with conventional technique, flattening filter-free model shortened the treating time with equivalent target dose and normal tissue dose. The median follow-up time is 19.4 mo. The 1-yr local control, regional control, distant control, progression free survival and overall survival rates were 96.8%, 96.8%, 83.9%, 77.4% and 96.8%. The most common side effects were radiation pneumonitis (29% grade 1, 3.2% grade 2) and chest pain (12.9% grade 1, 6.5% grade 2).

**Conclusion:**

The use of flattening filter-free model in SBRT for small lesions of NSCLC patients is safe and effective. Long time follow-up and additional studies are still needed to validate our conclusions.

肺癌在中国的发病率呈连年上升趋势，且是癌症相关死亡的首要原因^[1, 2]^。以2003年-2007年的肺癌死亡率为43.48/10万左右计算^[3]^，我国每年约有60万人死于肺癌。其中非小细胞肺癌（non-small cell lung cancer, NSCLC）占原发性肺癌总人数的75%-80%。

随着放疗技术的发展，立体定向放射治疗（stereotactic body radiation therapy, SBRT）的研究和临床应用日益广泛，特别是治疗NSCLC肺部小病灶的临床应用研究十分活跃，其结果也令人振奋。对于临床Ⅰ期NSCLC，予以生物等效剂量大于100 Gy的SBRT治疗，其3年局控率可达84.2%，3年及5年生存率为80.4%和70.8%^[4]^，与手术治疗相当^[5]^。

我科近十年开展了对肺部病灶的SBRT治疗，自2011年12月开始利用非均整模式加速器（TrueBeam, Varian Medical Systems）进行治疗，它可投射6兆伏及10兆伏的非均整X射线，最高剂量率分别达到1, 400 MU/min及2, 400 MU/min。均整器的使用是为了获得强度分布均匀的较大射野，用于常规治疗。非均整模式移除了加速器的射野均整器，提高了剂量率，为常规均整模式的4倍左右。非均整模式射野的剂量分布与常规模式有较大差别，可在治疗时间减少50%以上的情况下，完成单次大剂量的照射计划^[6-8]^。然而由于临床经验较少，国内暂无相关文献报道，非均整模式SBRT治疗的安全性和有效性尚待观察。笔者采用此技术治疗31例NSCLC肺部小病灶报告如下。

## 1 资料和方法

### 1.1 研究对象

选取T1-2N0M0的原发NSCLC，NSCLC术后肺内孤立复发转移，以及Ⅳ期肺内寡转移者。病例选择标准：经纤维支气管镜、穿刺活检病理或细胞学确诊的NSCLC；进行过完整的临床评估[病史、体检、血常规、血生化、尿常规、心电图、肺功能、胸部计算机断层扫描（computed tomography, CT）、浅表淋巴结B超、腹部B超、脑磁共振成像（magnetic resonance imaging, MRI）和同位素骨扫描]；年龄18岁以上；东部肿瘤合作组（Eastern Cooperative Oncology Group, EOCG）0分-2分；近6个月内体重下降不超过10%；通过CT可测量评估病灶；未接受过胸部放疗；无其他严重内科合并症；无重要器官的功能障碍，血常规、肝肾功能及心肺功能基本正常；Ⅳ期患者经化疗或靶向治疗后病情控制较稳定；理解并签署知情同意书。

自2011年12月-2013年12月，共有31例符合条件的NSCLC肺部小病灶患者。其中男性16例，女性15例。年龄50岁-83岁，平均年龄69岁。T1-2N0M0 NSCLC 15例，术后肺内孤立复发转移6例，Ⅳ期肺内寡转移者10例。中央型病变定义为发生在段支气管至主支气管的肺癌，即距离关键结构2 cm以内，包括气管1例，大血管3例，食管1例，心脏和心包各1例。其余24例患者病变为周围型。7例患者主观拒绝手术，8例患者因慢性阻塞性肺病（chronic obstructive pulmonary disease, COPD）等内科疾病无法耐受手术治疗，16例肺内复发转移癌患者不考虑手术治疗。患者一般情况见表 1。

**1:** 31例NSCLC肺部小病灶患者的临床资料 Clinical characteristics of 31 NSCLC patients with small lung lesions

Clinical characteristic	*n*	Percentage
Gender		
Male	17	54.8%
Female	14	45.2%
Age (yr)		
≤70	16	51.6%
>70	15	48.4%
Pathology type		
Squamous cell carcinoma	10	32.2%
Adenocarcinoma	18	58.1%
Large cell carcinoma	3	9.7%
Diameter of the lesions		
≤3 cm	20	64.5%
3 cm-5 cm	9	29.0%
>5 cm^*^	2	6.5%
Stage		
T1-T2N0M0	15	48.4%
Solitary pulmonary recurrence after surgery	6	19.4%
Stage Ⅳ with oligo metastasis	10	32.2%
ECOG performance status		
0	8	25.8%
1	21	67.7%
2	2	6.4%
Weight loss		
≤5%	26	83.9%
>5%	5	16.1%
Dose		
60 Gy/8 f	16	51.6%
48 Gy/4 f	15	48.4%
Reasons not taking surgery		
Refuse	7	22.6%
Not appropriate stage	16	51.6%
Complications	8	25.8%
^*^Two cases of >5 cm diameter were 5.1 cm and 5.3 cm. NSCLC: non-small cell lung cancer. ECOG: Eastern Cooperative Oncology Group.		

### 1.2 治疗方法

所有患者均采用调强放射治疗。首先行热塑模固定，所有患者均采取仰卧位，双手交叉抱肘置于前额。定位CT采用静脉增强螺旋扫描，层厚5 mm，扫描范围为环状软骨上缘至肝脏下缘。采用模拟定位机采集病例的呼吸周期运动图像，测量各个方向的呼吸运动幅度。靶区勾画：大体肿瘤靶区（gross tumor volume, GTV）包括CT上显示的原发或转移肿瘤，在肺窗中勾画；将肿瘤跟随呼吸运动的各方向位移幅度输入系统，外扩得到内在大体肿瘤靶区（internal gross tumor volume, IGTV）；将IGTV的范围沿三维方向外扩0.5 cm得到计划靶区（planning target volume, PTV）。如有阻塞性肺不张，需参考PET或MRI结果协助确定靶区边缘。处方剂量为IGTV 60 Gy/8 f或48 Gy/4 f，其中中央型病变选择单次剂量较小的60 Gy/8 f方案，周围型病变选择单次剂量大的48 Gy/4 f方案，生物有效剂量（biological effective dose, BED）均为105 Gy。重要组织器官受照射剂量均控制在可接受范围之内，如脊髓最大剂量点≤18 Gy，心脏≤30 Gy，双肺V20≤10%，食管≤27 Gy，气管及支气管≤30 Gy，大血管最大剂量点≤55 Gy。采用瓦里安Eclipse 10.0治疗计划系统，TrueBeam加速器非均整模式6兆伏X射线制定治疗计划。要求处方剂量至少包绕95%的靶区体积，处方剂量110%所包绕的体积不得超过靶区体积的1%。采用剂量体积直方图评估计划剂量分布。每次治疗前采用锥形束CT（cone beam computed tomography, CBCT）检验并校正目标靶区及关键结构的位置。

### 1.3 疗效及毒副反应评估

根据世界卫生组织（World Health Organization, WHO）与国际抗癌联盟制定的实体肿瘤客观疗效标准（Response Evaluation Criteria in Solid Tumors, RECIST）1.1版本，治疗后1个月和3个月分别复查胸部增强CT，并以3个月时的影像资料作为近期疗效的判定。CT上病灶常表现为团块、团块状纤维化、疤痕样纤维化等，我们测量实性成分的最长径线进行判定，疤痕样的纤维化则认为达到CR。远期疗效评价指标包括局部控制率（local control, LC）、区域控制率（regional control, RC）、远处转移控制率（distant control, DC）、无疾病进展率（progression free survival, PFS）、总生存率（overall survival, OS）。局部失败定义为CT证实的病变进展或PET提示SUV最大摄取值>5，或6个月后PET/CT上同一肺叶内出现新病灶^[9]^。区域复发指纵隔和/或锁骨上淋巴结转移。远处转移则指远隔淋巴结、对侧肺或其他器官（脑、骨、肝、肾上腺等）转移。对于可疑转移灶，强烈建议进行活检。无疾病进展时间定义为患者放疗开始日期至疾病进展日期。总生存时间定义为患者放疗开始日期至末次随访日期或死亡日期，在末次随访时仍存活者生存时间计算截尾值。毒副反应按美国国家癌症研究所（National Cancer Institute, NCI）制定的常用不良反应事件评价标准（Common Terminology Criteria for Adverse Events, CTCAE）3.0版本的分级进行评价。其中LC、RC、毒副反应为主要观察目标，DC、PFS、OS为次要观察目标。

### 1.4 统计学方法

所有患者治疗后需定期随访，2年内每3个月1次，2年-5年内每6个月1次。统计采用SPSS 13.0软件，采用*Kaplan*-*Meier*生存分析（*Log*-*rank*法）分别计算LC、RC。用*χ*^2^检验进行率的比较。以*P* < 0.05为差异有统计学意义。

## 2 结果

### 2.1 随访时间

末次随访时间为2014年12月，中位随访时间为19.4月，随访率100%。

### 2.2 治疗时间及剂量

非均整模式治疗的时间明显缩短。随机抽取照射剂量为48 Gy/4 f的8例患者，与既往我科采用常规均整模式治疗的8例相同剂量的病例比较，治疗时间由平均6.25 min（3.5 min-8 min）缩短至2.93 min（2 min-3.5 min），有统计学差异（*P*=0.005）。非均整模式在缩短治疗时间的同时，靶区剂量及各个正常组织的受量与常规均整模式无明显差异，如图 1所示。

**1:**
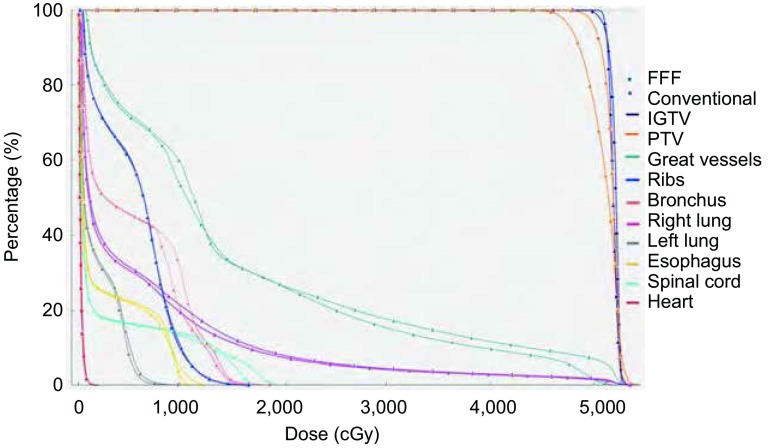
48 Gy/4 f方案非均整模式和常规均整模式的剂量体积直方图 Dose volumn histograms of flattening filter-free (FFF) model and conventional model with flattening filter of 48 Gy/4 f regimen. IGTV: internal gross tumor volume; PTV: planning target volume

### 2.3 近期疗效

以治疗后3个月复查的胸部增强CT为判定依据，全部患者中完全缓解（complete response, CR）10例，部分缓解（partial response, PR）15例，疾病稳定（stable disease, SD）6例，无疾病进展（progressive disease, PD）患者。总有效率（CR+PR）分别为25/31（80.7%）。

### 2.4 疾病进展和生存

随访满1年时，分别有1例、1例、5例患者出现局部复发、区域转移和远处转移。1例患者因疾病进展死亡。1年的LC、RC、DC率分别为96.8%、96.8%和83.9%（图 2A）。1年OS和PFS率分别为96.8%和77.4%（图 2B）。

**2:**
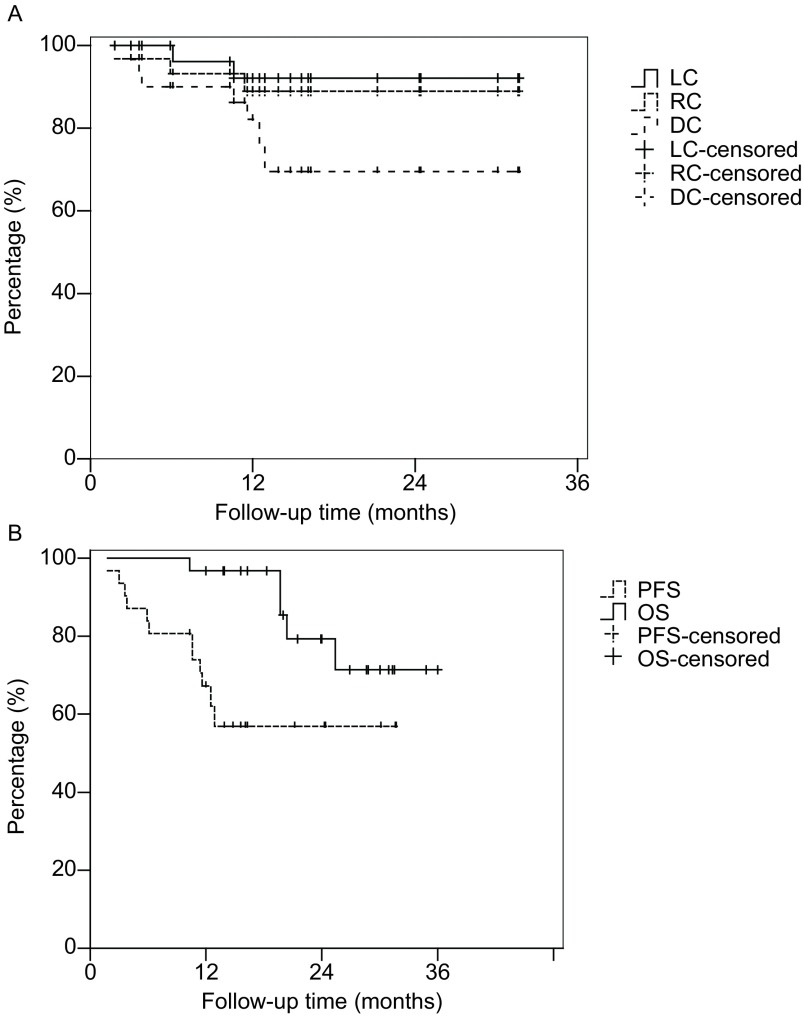
31例NSCLC行非均整模式大分割放疗的*Kaplan*-*Meier*分析(A) LC、RC和DC；(B) OS和PFS *Kaplan*-*Meier* analysis of 31 NSCLC patients receiving flattening filter-free model SRBT. A: Local control (LC), regional control (RC) and distant control (DC); B: Overall survival (OS) and progression free survival (PFS)

分别对LC、RC、DC、PFS、OS做了潜在相关性的分析，影响因子如下：年龄、性别、ECOG评分、病理类型、病变位置、病变大小、原发或复发转移病变、放疗剂量和有无体重下降。其中PFS、LC、RC、DC均未找到独立相关因素。仅分期及病变大小（T）倾向对OS有意义，*P*值分别0.051和0.054。

进而我们对不同分期的患者做了亚组分析。随访满1年时，15例T1-2N0M0原发性NSCLC中1例出现局部复发，无死亡病例。6例术后孤立复发患者中出现2例远处转移，无死亡病例。10例Ⅳ期肺内寡转移者中有1例区域复发，3例广泛转移；其中1例死亡（表 2）。

**2:** 不同亚组的1年局部、区域、远处转移控制率和总生存率 Subgroup analysis of one year LC, RC, DC and OS rates

Subgroup	*n*	LC	RC	DC	OS
T1-T2N0M0	15	14 (93.3%)	15 (100.0%)	15 (100.0%)	15 (100.0%)
Solitary pulmonary recurrence after surgery	6	6 (100.0%)	6 (100.0%)	4 (66.7%)	6 (100.0%)
Stage Ⅳ with oligo metastasis	10	10 (100.0%)	9 (90.0%)	7 (70.0%)	9 (90.0%)

### 2.5 毒副反应

6例患者出现1度血液学毒性，包括3例白细胞减低，1例中性粒细胞减低，2例血小板减低，未予药物治疗，1周-2周内恢复正常。乏力及纳差、恶心等消化道副反应共6例，给予改善食欲、营养支持等治疗后好转。3例1度及1例2度放射性食管炎患者，均为中央型病变，予对症治疗，放疗后2周-4周恢复。9例患者1度放射性肺炎，均为复查CT时所见病变周围有少许斑片影或絮状影，均无咳嗽、发热、呼吸困难等症状，继续随访观察后大部分吸收消失，无明显纤维化表现。1例患者放疗后因低热、活动时气促就诊，胸部CT新见斑片影，考虑2度放射性肺炎，经激素治疗后痊愈。共6例出现胸痛，2例2度胸痛者服用止痛药对症治疗，其中1例经核磁检查诊断为肋骨骨折，无错位，约2月后疼痛缓解消失。大咯血、臂丛神经损伤、心律失常等副反应均未在随访中观测到（表 3）。

**3:** 31例患者的毒副反应 Adverse events of 31 NSCLC patients with small lung lesions

Adverse events	Grade Ⅰ	Grade Ⅱ	Grade Ⅲ	Grade Ⅳ
Bone marrow suppression	6 (19.4%)	-	-	-
Fatigue	2 (6.5%)	1 (3.2%)	-	-
Gastrointestinal side effects	4 (12.9%)	-	-	-
Radiation esophagitis	3 (9.7%)	1 (3.2%)	-	-
Radiation pneumonitis	9 (29.0%)	1 (3.2%)	-	-
Chest pain	4 (12.9%)	2 (6.5%)	-	-
Skin reaction	3 (9.7%)	-	-	-

分别将病变大小、病变位置、单次放疗剂量、分割次数与上述副反应进行相关性分析，均未见独立相关因素。

## 3 讨论

肿瘤增殖快、倍增时间短，总疗程延长会导致肿瘤局部控制率及生存率降低。因此从技术角度上讲，单次剂量大、分割次数少的SBRT疗法，可显著提高肿瘤生物效应剂量，降低对正常组织器官的损伤，进而提高疗效。在NSCLC中，SBRT适用于早期患者，以及不适合全身化疗伴有肺内小转移灶的患者。既往的研究多采用常规均整模式，即经射野均整器后射线剂量率较低的模式。随着技术的进步，非均整模式去除射野均整器后，射线剂量率得到大幅提高。而这种方式在NSCLC临床治疗中的疗效及毒副反应如何，便是本文关注并尽力回答的问题。

定位CT我们采用了5 mm层厚，但每一次治疗前均拍摄CBCT校准治疗位置，保证其误差在3 mm之内。通过模拟定位机连续采集呼吸周期图像，测量各个方向的呼吸运动幅度，进而在勾画靶区时由GTV及呼吸动度得到IGTV。未外扩CTV，主要是考虑大分割放疗的等效生物剂量已较高，可达到控制肿瘤的目的；靶区体积不宜过大，以减少射线相关损失。剂量上我们予以周围型48 Gy/4 f，而中央型病变因周围的危及器官耐受剂量有限，给予了单次剂量较小的60 Gy/8 f方案。

我们的结果表明，非均整模式比较均整模式大幅缩短了治疗时间，靶区剂量相当，且未增加正常组织受量。中位随访19.4个月。治疗后3个月全部患者的客观缓解率达到80.7%。之后部分PR患者的病灶有继续缩小的趋势，截至末次随访CR患者均未出现局部复发。1年的LC、RC、DC、PFS和OS率分别为96.8%、96.8%、83.9%、77.4%和96.8%。毒副反应可耐受或治疗后好转。

放射生物学方面，剂量率是否直接影响细胞死亡一直存在争议。剂量率效应是指随着剂量率的提高，细胞存活下降。但最近的临床前研究提出与之不同的观点。Verbakel等^[10]^比较了三种不同细胞系的克隆形成，分别使用高剂量率和常规均整剂量率的射线，结果并无差异。Sørensen等^[11]^在5.01-29.91 Gy/min的区间内并未证明剂量率效应。同样，Ling等^[12]^也指出剂量率效应与时间相关，而并非单纯与剂量率有关。

临床应用上，既往对NSCLC常规均整模式SBRT的研究较多，且病例数较多，随访时间较长。Onishi等^[4]^报道日本14家医院高分次剂量治疗257例各种原因不可手术的早期NSCLC的结果：总有效率为86.8%。215例BED≥100 Gy的患者5年生存率为70.8%，明显高于42例BED < 100 Gy的5年生存率30.2%。荷兰的Suzuki等^[13]^报道了两个中心接受常规模式SBRT治疗的383例早期NSCLC患者，BED在84 Gy-102 Gy，3年的生存率为60%，局部控制率为84%-93%。其中主观拒绝手术的患者比因合并其他疾病不能手术者更能获益。而非均整模式应用于NSCLC的临床报道较少。Navarria等^[14]^报道了自2006年-2011年行SBRT治疗的132例Ⅰ期NSCLC患者，其中先期入组的86例是常规均整模式，后入组的46例采用了非均整模式。3个月时有效率达85%。中位随访16个月，非均整模式的1年局部控制率为100%，优于常规均整模式的92.5%（*P*=0.03）。但两组的一般特点存在差异，非均整组的病灶直径大于常规模式组，而平均年龄较小，且研究随访时间较短。本研究回顾了我科31例采用非均整模式SBRT治疗的NSCLC肺部小病灶患者，初步结果与前述常规均整模式的临床研究相近，也与我科既往报道的^[15]^10例采用常规均整模式SBRT治疗早期NSCLC的结果相近（1年的局部控制率和生存率均为100%），说明非均整模式的近期局部及区域控制情况不劣于均整模式SBRT。

常规均整模式SBRT毒副反应方面，Onishi等^[4]^报道的257例结果显示：2级以上的放射性肺炎发生率为5.4%。Baumann等^[16]^回顾性分析的138例患者2级以上的肺部并发症发病率为1%。Lucas等^[17]^报道的81例接受SBRT治疗的Ⅰ期或Ⅱ期无淋巴结转移患者，无3级-4级的副反应，1级放射性肺炎发生率为4%，6.2%的患者出现胸痛。非均整模式SBRT治疗的副反应亦较轻，与上述研究相近。Navarria等^[14]^观察的46例患者中，有8例（17.4%）出现1级-2级放射性肺炎，2例（4%）出现3级肺炎。本研究中仅出现1例2级放射性肺炎，2例2级胸痛，另有少数1级反应，治疗后均好转。无严重的治疗相关不良事件，安全性及耐受性较好。需要注意的是，并非所有发生急性副反应（治疗后3月以内发生）的患者都会出现慢性副反应（超过3个月发生），也并非所有出现了慢性副反应的患者都曾经有急性副反应。对于慢性副反应的观察还需要长时间的监测。

此外，临床前研究表明^[18, 19]^非均整射线在低剂量区的边缘处产生更少的光子污染，且MLC多叶光栅泄露更少。因其能减少约70%的光子散射污染，有人提出非均整模式可能减少二次肿瘤的发生率^[20]^。提示非均整治疗模式可能有潜在获益，但结果尚待临床证实。

本研究的初步结果提示相比常规均整模式，使用非均整模式大分割放射治疗技术治疗肺部小肿瘤的安全性、有效性与之相近。但作为回顾性研究，病例数较少，随访时间较短，仍需长期、动态观察。
